# Genomic Analysis of Two Histamine-Producing Strains Isolated from Yellowfin Tuna

**DOI:** 10.3390/foods14091532

**Published:** 2025-04-27

**Authors:** Yazhe Wang, Di Wang, Shengjun Chen, Gang Yu, Zhenhua Ma, Ya Wei, Chunsheng Li, Yueqi Wang, Chaoming Shen, Yongqiang Zhao

**Affiliations:** 1 College of Food Science & Technology, Shanghai Ocean University, Shanghai 201306, China; m220301027@st.shou.edu.cn; 2 Key Laboratory of Aquatic Product Processing, Ministry of Agriculture and Rural Affairs, South China Sea Fisheries Research Institute, Chinese Academy of Fishery Sciences, Guangzhou 510300, China; chenshengjun@scsfri.ac.cn (S.C.); weiya@scsfri.ac.cn (Y.W.); lichunsheng@scsfri.ac.cn (C.L.); wangyueqi@scsfri.ac.cn (Y.W.); 3 Key Laboratory of Efficient Utilization and Processing of Marine Fishery Resources of Hainan Province, Sanya Tropical Fisheries Research Institute, Sanya 572426, China; gyu0928@163.com (G.Y.); zhenhua.ma@scsfri.ac.cn (Z.M.); 4 Beihai Product Quality Testing Institute, Beihai 536000, China; chaoming_shen@126.com

**Keywords:** *Morganella psychrotolerans*, *Morganella morganii* subsp. *Sibonii*, spoilage, whole genome sequencing

## Abstract

Psychrotrophic *Morganella* spp. is a typical histamine producer commonly found in seafood, exhibiting a high histamine-producing capacity. In this study, two strains of *Morganella* (GWT 902 and GWT 904) isolated from yellowfin tuna were subjected to phenotypic and genotypic characterization. Phenotypic analysis reveals differences in growth temperature, NaCl tolerance, and D-galactose fermentation capacity between the two strains. Notably, the histamine production capacity of GWT 902 is significantly higher than that of GWT 904 at 4 °C. The complete genome sequences of strains GWT 902 and GWT 904 were sequenced, identifying GWT 902 as *Morganella psychrotolerans* and GWT 904 as *Morganella morganii* subsp. *sibonii*. Genomic analysis confirms the presence of histidine decarboxylase gene clusters (*hdcT1*, *hdc*, *hdcT2*, *hisRS*) in both strains, and sequence alignment shows that the amino acid sequence similarity of histidine decarboxylase encoded by the *hdc* gene was 95.24%. Gene function analysis further identified genes associated with putrescine biosynthesis, sulfur metabolism, lipase and protease secretion, and detected key genes in quorum sensing (QS), stress adaptation, and antibiotic resistance. This study provides valuable insights into the taxonomic analysis of psychrotrophic *Morganella* spp. and contributes to the development of efficient strategies for preventing histamine formation in seafood.

## 1. Introduction

Seafood is favored by consumers for its delicious taste, nutrient richness, and high content of unsaturated fatty acids, and serves as an important source of protein. However, during processing and storage, seafood proteins degrade into free amino acids, which are subject to decarboxylation by microbial amino acid decarboxylases, leading to the formation of biogenic amines (BAs) [1]. Among BAs, histamine is the most toxic to the human body, and excessive intake may induce toxic reactions, including urticaria, nausea, vomiting, diarrhea, headache, and convulsions [2]. The Food and Drug Administration (FDA) has established a maximum allowable level of 50 ppm for histamine in fish products [3].

Histamine accumulation is primarily driven by histamine-producing bacteria, which convert free histidine into histamine via histidine decarboxylase enzymes [4]. *Morganella* spp. are common histamine-producing bacteria in seafood [5,6]. This genus includes *Morganella morganii* and *Morganella psychrotolerans*, with *M. morganii* further divided into two subspecies: *M. morganii* subsp. *sibonii* and *M. morganii* subsp. *morganii*. Rémy et al. [7] proposed a modified taxonomy, with the addition of a new species represented by a unique strain, suggesting that the taxonomy of this genus continues to improve. Both *M. morganii* and *M. psychrotolerans* have been isolated from various seafood products and exhibit a high capacity for histamine production [8,9]. Notably, *M. psychrotolerans* can produce toxic levels of histamine even at 0 °C, posing a significant threat to seafood safety during cold chain transportation [10].

Recent advances in high-throughput sequencing technologies have enabled whole genome sequencing (WGS) of bacteria. Compared to traditional typing techniques, WGS technology has greatly advanced bacterial species identification studies [11]. Furthermore, exploring bacterial metabolism pathways through gene mining is essential for gaining insights into bacterial behaviors, such as spoilage, stress resistance, and drug resistance [12,13]. Previous studies have conducted pan-genomic analyses of *M. morganii*, revealing differences in the distribution of virulence genes between the two subspecies [14]. However, a comprehensive genomic analysis of *M. psychrotolerans*, as well as comparative genomic studies between *M. psychrotolerans* and *M. morganii* have not been reported yet.

In this study, we focus on two psychrotrophic *Morganella* strains isolated from yellowfin tuna. We analyzed their characteristics, histamine production, and genomic features to expand our understanding of their amino acid metabolism, quorum sensing (QS) system, stress adaptation, and antibiotic resistance. This research offers novel insights into the genomic information of psychrotrophic *Morganella* spp. and gives potential targets for inhibiting histamine formation in *Morganella* spp.

## 2. Materials and Methods

### 2.1. Strains Isolation and Identification

The yellowfin tuna used in this study was purchased from a supermarket in Guangzhou, China. Next, 25 g of fish samples were aseptically homogenized in 225 mL of sterile phosphate-buffered saline (0.01 M PBS, pH 7.2) and the isolation of *Morganella* spp. bacteria was conducted according to our previous studies [15]. The isolated bacteria were preserved using Microbank^TM^ beads (Pro Lab Diagnostics, Richmond Hill, ON, Canada).

All strains isolated from fish samples were identified using sequence analysis of the 16S rDNA [16]. Genomic DNA was extracted from strains using Bacterial Genomic DNA Extraction Kit (Genstone Biotech, Beijing, China) according to the manufacturer’s instructions. The primers used were 27F (5′-AGAGTTTGATCCTGGCTCAG-3′) and 1492R (5′-TACGACTTAACCCCAATCGC-3′). Each 50 µL PCR reaction mixture contained 2 μL of template DNA (20 ng/μL), 2.5 µL of each primer (10 µM), 25 µL of 2X PCR Bestaq MasterMix (ABMgood, Vancouver, BC, Canada), and 18 µL of H_2_O. The PCR conditions were as follows: initial denaturation at 95 °C for 5 min, followed by 30 cycles of 94 °C for 45 s, 55 °C for 45 s, and 72 °C for 45 s, with a final extension at 72 °C for 1 min. The PCR products were purified from gel by QIAquick Gel Extraction Kit (Beijing Bestopbio Technology Co. Ltd., Beijing, China). The recovered PCR products were then sequenced by ABI3730XL sequencer (ABI, Foster City, CA, USA). The sequencing results were compared with NCBI database using BLAST (version 2.15.0), and the phylogenetic trees based on 16S rDNA gene sequences were constructed using the neighbor-joining tree method in MEGA 11 software.

### 2.2. Phenotypic Characterization

Two isolated strains were identified as *Morganella* spp. by 16S rDNA sequence analysis and named GWT 902 and GWT 904, respectively. Physiological and biochemical experiments, including 2 °C and 4 °C incubation, 8.5% NaCl incubation, and D-galactose fermentation experiments were performed to characterize the strains [17].

### 2.3. Determination of Histamine Production

The production of histamine was measured for two strains. Each stain was incubated in TSB with 1% L-histidine hydrochloride added (pH 6.0) at a concentration of approximately 5.0 × 10^5^ CFU/mL. After incubation at 20 °C for 60 h and 4 °C for 8 days, histamine contents of the culture were determined with the histamine test kit (Kikkoman Biochemifa Company, Tokyo, Japan), according to the manufacturer’ s instructions. The data were analyzed using SPSS 26 (IBM, Chicago, IL, USA). The independent sample *t*-test was employed for comparisons between two strains. *p* < 0.05 was considered significant.

### 2.4. Genome Sequencing and Assembly

Strains were cultured in sterile TSB medium at 25 °C to the middle of logarithmic growth. Bacterial cells were collected by centrifugation for 10 min (10,000× *g*, 4 °C) and washed twice with sterilized phosphate-buffered saline (PBS). Genomic DNA was extracted using the Wizard^®^ Genomic DNA Purification Kit (Promega, Madison, WI, USA) according to the manufacturer’s instructions. Purified genomic DNA was quantified with a NanoDrop2000 spectrophotometer. High-quality DNA (OD260/280 = 1.8–2.0, >20 μg) was used for subsequent analysis.

The whole genome of strains was sequenced using a combination of the Illumina NovaSeq 6000 and Nanopore DNA sequencing platforms. For Illumina sequencing, ≥1 μg of genomic DNA was fragmented into 400–500 bp fragments using a Covaris M220 Focused Acoustic Shearer. Libraries for sequencing were constructed using the NEXTflex™ Rapid DNA-Seq Kit (Bioo Scientific, Austin, TX, USA), with adapter-ligated products enriched through PCR. Paired-end sequencing (2 × 150 bp) was performed using the Illumina NovaSeq 6000 platform. For Nanopore sequencing, library construction was carried out using the SQK-LSK109 kit, and multiplexing was performed with the EXP-NBD104 barcoding kit following the manufacturer’s protocols. Sequencing was completed using the R9.4.1 flow cell and MinION device. Base calling and demultiplexing were carried out using Guppy v.3.1.5 (ONT). The assembly of the complete genome sequence was executed through the utilization of a combination of Nanopore and Illumina reads. Raw sequencing data were stored in FASTQ files, containing read sequences and quality information. Clean data were generated through quality control using FASTQ (https://github.com/OpenGene/fastp (accessed on 10 March 2024)). Read assembly into contigs was performed using Unicycler v0.4.8 [18]. After manual verification and circularization, the final genome sequence with seamless chromosomes and plasmids was obtained. Nanopore assembly results were further corrected for errors using Illumina reads through Pilon v1.22 software [19].

### 2.5. Gene Function Annotation

GeneMarkS version 4.3 [20] was employed for predicting coding DNA sequences (CDSs). tRNA and rRNA sequences were predicted using tRNA-scan-SE version 2.0.12 [21] and Barrnap version 0.9 (https://github.com/tseemann/barrnap (accessed on 10 March 2024)), respectively. Genomic circle mapping was performed using CGView [22]. The predicted CDSs were annotated from the databases such as Gene Ontology (GO) (http://www.geneontology.org/ (accessed on 12 March 2024)), Clusters of Orthologous Groups (COG) (http://eggnog.embl.de/ (accessed on 12 March 2024)), and the Kyoto Encyclopedia of Genes and Genomes (KEGG) (http://www.genome.jp/kegg/ (accessed on 12 March 2024)) using the sequence alignment tool BLAST+ (http://ftp.ncbi.nlm.nih.gov/blast/executables/blast+/2.3.0/ (accessed on 12 March 2024)). The Comprehensive Antibiotic Resistance Database (CARD) (http://arpcard.Mcmaster.ca (accessed on 12 March 2024)) was used for drug resistance analysis.

### 2.6. Average Nucleotide Identity (ANI) Analysis

ANI is the average base similarity between homologous segments of a genome, an indicator for comparing the relatedness of two genomes at the nucleotide level, and is widely used for taxonomic identification of bacteria with sequenced genomes [23]. The five genome sequences of *M. psychrotolerans* JCM 16473 (ASM3952365v1), *M. psychrotolerans* DI-20 (ASM167605v1), *M. psychrotolerans* DI-3 (ASM167622v1), *M. morganii* ATCC 25830 (ASM609445v1), and *M. sibonii* DSM 14850 (ASM4056024v1) were downloaded from NCBI. The ANI values were analyzed using the JspeciesWS online service (http://jspecies.ribohost.com/jspeciesws/ (accessed on 16 January 2025)).

### 2.7. Comparative Analysis of Homologous Genes of GWT 902 and GWT 904

OrthoFinder was used to conduct pairwise genomic comparative analysis of GWT 902 and GWT 904 [24]. The common genes and unique genes were analyzed, and the unique genes were analyzed with COG and KEGG annotation.

## 3. Results and Discussion

### 3.1. Strains Identification Analysis

**Figure 1:**
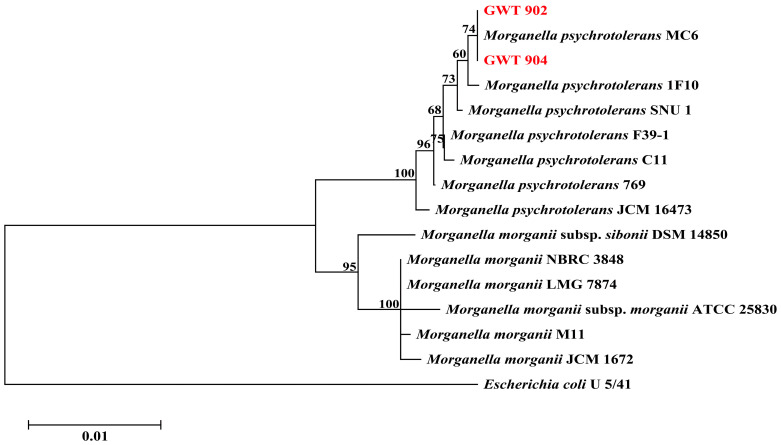
Phylogenetic tree of GWT 902 and GWT 904 based on 16S rDNA gene sequences.

As shown in Figure 1, the phylogenetic tree of strains GWT 902 and GWT 904 was constructed based on the 16S rDNA gene sequences. The two strains were closely related to *M. psychrotolerans* MC6. The results of the 16S rDNA gene sequences show that GWT 902 and GWT 904 are *M. psychrotolerans*.

The physiological and biochemical characteristics of two isolated strains are shown in Table 1. GWT 902 is capable of growth at 2 °C but cannot grow in 8.5% NaCl or ferment D-galactose. In contrast, GWT 904 cannot grow under 4 °C and it can grow in 8.5% NaCl and fermented D-galactose. There are differences between *M. psychrotolerans* and *M. morganii* in terms of growth temperature, NaCl tolerance, and D-galactose fermentation [17]. Based on the characteristic tests, we suspected that strain GWT 904 is *M. morganii*. However, this is inconsistent with the results of the 16S rDNA analysis. Therefore, we sequenced the whole genome of both strains for further species identification analysis.

### 3.2. Histamine Determination

The results of histamine content are shown in Table 2. The histamine contents of GWT 902 and GWT 904 were 4807.93 mg/L and 5590.27 mg/L, respectively, after incubation at 20 °C for 60 h. After incubation at 4 °C for 8 days, the histamine contents of GWT 902 and GWT 904 were 4708.17 mg/L and 3731.57 mg/L, respectively. High histamine-producing bacteria are defined as those capable of producing more than 1000 mg/L histamine in tuna fish infusion broth or tryptic soy broth (TSB) supplemented with 2% histidine after being cultured at temperatures above 15 °C for 24 to 48 h [25]. The histamine production observed in both strains at 20 °C after 60 h was higher than 1000 mg/L, suggesting that both strains possess a high capacity for histamine formation. Furthermore, the results show that there was a difference between GWT 902 and GWT 904 in histamine production capacity, with GWT 902 exhibiting a stronger capacity under low-temperature conditions.

### 3.3. Genome Features of GWT 902 and GWT 904

**Figure 2:**
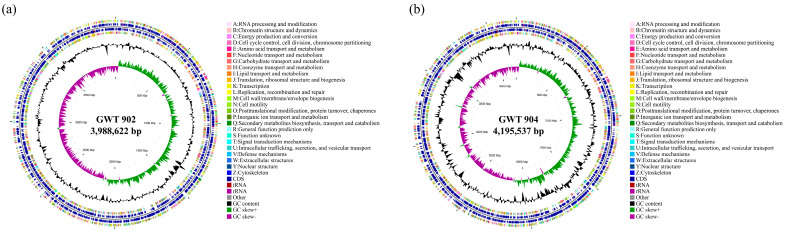
Circular genome map of GWT 902 (**a**) and GWT 904 (**b**). Circles are numbered from the outermost (first) to the innermost (seventh) circle and include the following features: coding DNA sequences (CDS) on forward and reverse chains, with different colors based on clusters of orthologous groups of proteins (COGs) categories (first and fourth circles); CDS, rRNA, and tRNA on forward and reverse chains (second and third circles); GC content (fifth circle); GC-SKEW (sixth circle); genome size (seventh circle).

The whole genome sequences were submitted in GenBank with accession numbers PRJNA1222167 (for GWT 902) and PRJNA1222168 (for GWT 904). The circular maps of the genome are shown in Figure 2, and detailed genomic characteristics are listed in Table 3. The complete genome sequence of GWT 902 was 3,990,716 bp in length, with a G + C content of 47.97%, which contained 3608 coding genes and predicted the number of 22 rRNAs and 77 tRNAs; The complete genome sequence of GWT 904 was 4,200,263 bp in length, with a G + C content of 50.39%, which contains 3946 coding genes and predicts 22 rRNAs and 81 tRNAs.

### 3.4. ANI Analysis

**Figure 3:**
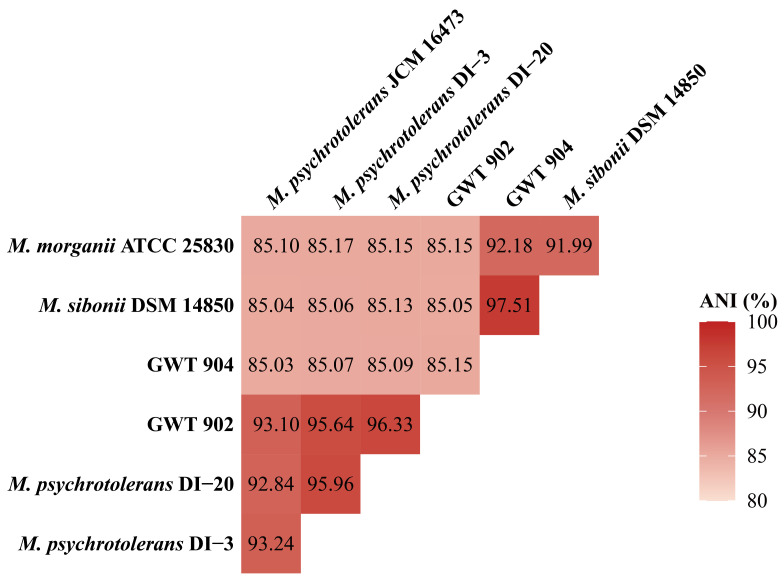
Pairwise comparison of the average nucleotide identity (ANI) of the genomes. The heat map shows the pairwise ANI as determined for the GWT 902, GWT 904, and several *Morganella* strains. The color scale is shown to the right of the heat map.

ANI analysis based on whole genome sequence is an accurate and effective method for bacterial identification, and the usual threshold for species classification is 95% [23,26]. As shown in Figure 3, the ANI values of GWT 902 with *M. psychrotolerans* DI-20 and *M. psychrotolerans* DI-3 are 96.33% and 95.64%, respectively. The results indicate that GWT 902 belongs to *M. psychrotolerans*. The ANI value between GWT 904 and *M. sibonii* DSM 14850 is 97.51%, which proves that strain GWT 904 has a higher homology with *M. sibonii*.

### 3.5. Functional Annotation of GWT 902 and GWT 904

**Figure 4:**
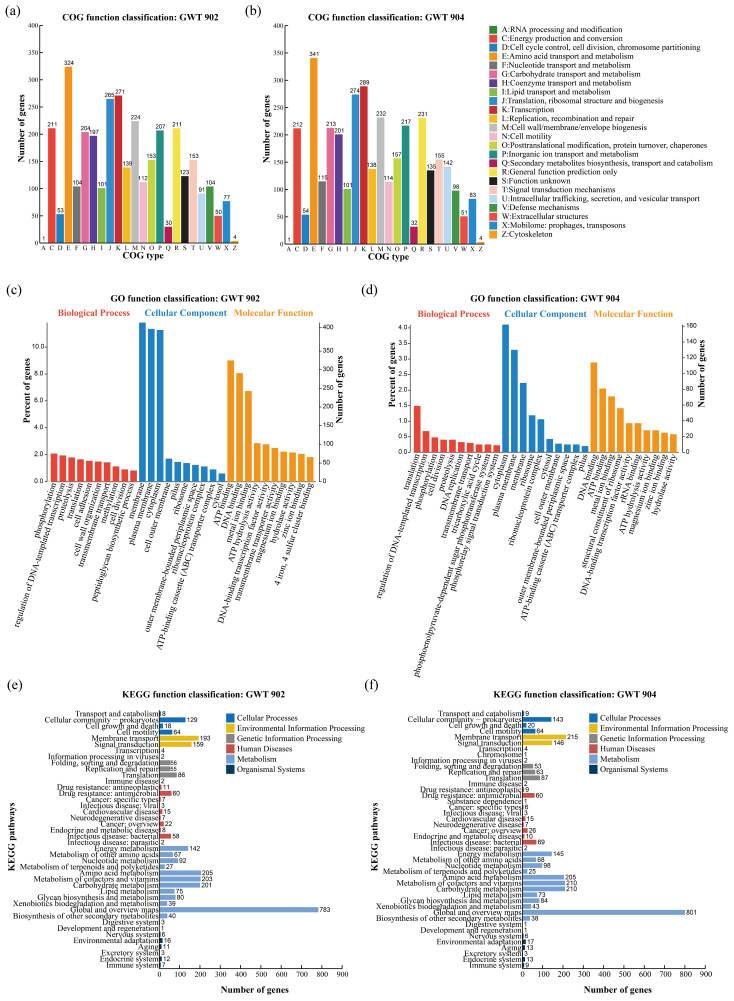
Whole-genome sequence analysis of functional categories of GWT 902 and GWT 904 annotated by clusters of orthologous groups (COG) (**a**,**b**); gene ontology (GO) (**c**,**d**); Kyoto encyclopedia of genes and genomes (KEGG) (**e**,**f**).

There are 3032 and 3184 coding genes annotated to 24 COG functional classifications for GWT 902 and GWT 904, respectively. As shown in Figure 4a,b, the COG annotation results were similar for both strains, with more genes annotated to E: amino acid transport and metabolism, K: transcription and J: translation, ribosome structure, and biosynthesis. GWT 902 and GWT 904 had 324 and 341 genes annotated to E: amino acid transport and metabolism, 271 and 289 genes annotated to K: transcription, 265 and 274 genes annotated to J: translation, ribosome structure, and biosynthesis, respectively.

A total of 2814 and 873 coding genes of GWT 902 and GWT 904 were classified into three functional categories by GO analysis, respectively. The top 10 GO terms for each category are shown in Figure 4c,d. The genes of two strains were most associated with molecular function (2237; 692), followed by biological process (1676; 563) and cellular component category (1554; 520). Within the molecular function category, the most abundant classifications were ATP binding (GO: 0005524), DNA binding (GO: 0003677), and metal ion binding (GO: 0046872). Genes related to cellular components were primarily associated with membrane (GO: 0016020), plasma membrane (GO: 0005886), and cytoplasm (GO: 0005737). In the biological process category, GWT 902 had more genes annotated to phosphorylation (GO: 0016310), while GWT 904 had more genes annotated to translation (GO: 0006412).

The KEGG database provides a systematic understanding of the biological functions of genes, such as metabolic pathways, genetic information transfer, cytological processes, and other complex biological processes [27,28]. A total of 2862 and 3014 coding genes are annotated to KEGG metabolic pathways in GWT 902 and GWT 904, respectively (Figure 4e,f). The metabolism involves the most genes; in addition to the global and overview pathways, the most abundant metabolism pathways in the genomes of both strains were amino acid metabolism, carbohydrate metabolism, cofactor and vitamin metabolism, containing more than 200 genes in each pathway. In addition, more genes were annotated to environmental information processing pathways including signal transduction and membrane transport. Genes were mainly associated with the two-component signal transduction system and ABC transporter proteins.

### 3.6. Comparative Analysis of Homologous Genes of GWT 902 and GWT 904

**Figure 5:**
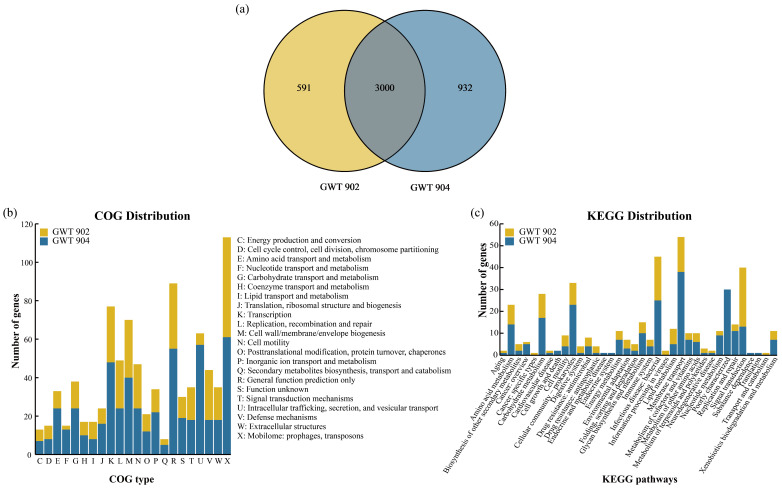
(**a**) Venn diagram depicting the common and unique homologous genes between GWT 902 and GWT 904. (**b**) COG and (**c**) KEGG distribution of unique genes of GWT 902 and GWT 904.

Figure 5a illustrates the common and unique homologous genes of GWT 902 and GWT 904. A total of 4523 genes were identified in two strains, with 3000 genes being common, and 591 and 932 genes unique to GWT 902 and GWT 904, respectively. Homologous genes, which have evolved from common ancestral genes in different species, can be categorized into orthologous and paralogous genes. Orthologous genes often share similar biological functions [29]. The high percentage of common genes suggests a close phylogenetic relationship between the two strains. Conversely, the presence of unique genes indicates that the strains may have undergone distinct adaptive evolutionary processes [30,31].

The unique genes of the two strains were annotated using COG and KEGG database (Figure 5b,c). In the COG annotation analysis, 314 and 462 unique genes were annotated to 22 COG classifications in GWT 902 and GWT 904, respectively. More unique genes of the two strains were annotated to X: Mobilome: prophages, transposons. It is reported that mobile genetic elements are responsible for the movement of drug resistance determinants and virulence factors between microorganisms [32]. In addition, GWT 904 exhibited a significantly higher number of unique genes in the category of U: Intracellular trafficking, secretion, and vesicular transport compared to GWT 902, particularly in the COG3468 components (autotransporter adhesin AidA). The autotransporter adhesins possess diverse functions that facilitate bacterial colonization, survival, and persistence [33], suggesting that GWT 904 may possess stronger fitness and pathogenic potential. In the KEGG annotation analysis, 255 and 405 unique genes were annotated to KEGG in GWT 902 and GWT 904, respectively. GWT 902 had more genes annotated to signal transduction, mainly involved in the two-component systems, which is of great significance for bacterial adaptation to environmental changes, drug resistance, and virulence factor production [34,35,36]. While GWT 904 contained more unique genes related to membrane transport, most of these were associated with bacterial secretion systems and ABC transporters. The differences in environmental information processing between GWT 902 and GWT 904 reflect their distinct adaptations to environmental changes. In conclusion, the comparative homologous genome analysis provided new insights into the differences between the genomes of the two strains.

### 3.7. Histamine Metabolism

Histidine decarboxylase (HDC) is the key enzyme catalyzing the conversion of histidine to histamine. As shown in Table 4, the *hdc* gene, which encodes HDC, was identified in both strains. Protein sequence alignment reveals that the HDC enzymes from both strains consist of 378 amino acids, sharing 95.24% sequence identity. It has been reported that HDC in *M. morganii* is a pyridoxal 5′-phosphate (PLP)-dependent enzyme, whose conserved lysine residues within the PLP binding site form a stable internal aldehyde-amine structure with PLP cofactors, which is essential for enzyme activity [37,38]. Both strains retain this essential catalytic lysine residue. However, differences in various amino acid residues might influence protein stability or substrate binding affinity, potentially modulating enzymatic activity. Such molecular differences may account for the observed phenotypic differences in histamine production between the two strains (Table 2). Furthermore, *hdcT1*, *hdcT2*, and *hisRS* were identified in GWT 902 and GWT 904. The *hdcT1* and *hdcT2* encode putative histidine/histamine antiporters, and *hisRS* encodes histidyl-tRNA synthetase. *hdcT1*, *hdc*, *hdcT2,* and *hisRS* constitute the histidine decarboxylase gene cluster, which is critically involved in histamine formation in histamine producers [4,38]. The identification of the histidine decarboxylase gene cluster offers valuable insights into the genetic basis of histamine production in psychrotrophic *Morganella* spp. This understanding enables us to screen for potential inhibitors of histamine formation based on computational biology.

### 3.8. Putrescine Metabolism

Putrescine is a major contributor to seafood spoilage and the associated unpleasant odors. As shown in Table 4, genes related to putrescine production, including *speC*, *speA,* and *speB*, which encodes ornithine decarboxylase, arginine decarboxylase, and agmatinase were identified in GWT 902 and GWT 904. *puuP* gene encoding putrescine importer and *pot* genes related to putrescine transportation were also identified in both strains. Ornithine decarboxylase is a key enzyme in the production of putrescine, and *speC* has been identified in *M. sibonii* isolated from cheese [39]. The *speC* gene was identified in GWT 902 and GWT 904, suggesting that these two strains may be able to produce putrescine.

### 3.9. Sulfur Metabolism

Sulfur metabolism is a key metabolic pathway that produces hydrogen sulfide (H_2_S) and off-odors in fish products [40]. A series of *cys* genes (*cysQ*, *cysW*, *cysU*, *cysA*, *cysM*, *cysP*, *cysE*, *cysK*, *cysZ*) involved in sulfur metabolism were identified in GWT 902 and GWT 904 (Table 4), which encodes 3′(2′), 5′-bisphosphate nucleotidase [EC:3.1.3.7], sulfate/thiosulfate transport system components, sulfate/thiosulfate transport system ATP-binding protein [EC:7.3.2.3], cysteine synthase [EC:2.5.1.144], sulfate/thiosulfate transport system substrate-binding protein, serine O-acetyltransferase [EC:2.3.1.30], cysteine synthase [EC:2.5.1.47], and sulfate transport protein. *cysM* has been reported as a critical gene in sulfur metabolism in *Shewanella baltica* and *Shewanella putrefaciens*, playing an essential role in bacterial spoilage potential. However, the *cysI* and *cysJ* genes, which encode sulfite reductase [EC:1.8.1.2], were only identified in GWT 904. Sulfite reductase catalyzes the reduction of sulfites to H_2_S. In addition, *tauD* gene encoding taurine dioxygenase [EC:1.14.11.17] [41], *sqr* gene encoding sulfide: quinone oxidoreductase, as well as genes related to thiosulfate (*glpE*, *sseA*), tetrathionate reductase subunits (*ttrA*, *ttrC*, *ttrB*), and sulfite dehydrogenase (quinone) subunit (*soeC*) were also identified in two strains [42,43]. Notably, sulfur metabolism plays an important role in bacterial energy metabolism and antioxidant defense [44]. The identification of sulfur metabolism-related genes in this study demonstrates that the GWT 902 and GWT 904 strains may possess strong sulfur metabolic capabilities. This capacity not only directly influences the production of H_2_S but may also indirectly affect various metabolic pathways in bacteria by modulating energy metabolism and antioxidant defense processes, providing a genetic foundation for a more comprehensive understanding of the mechanisms underlying bacterial spoilage in seafood.

### 3.10. Lipase and Protease

Lipase can catalyze the hydrolysis of lipids in aquatic products to produce free fatty acids, glycerol, and other metabolites that accelerate spoilage of aquatic products [45]. As shown in Table 4, genes encoding lipase, lipoyl (octanoyl) transferase, lysophospholipase, and esterase were found in two strains. The extracellular proteases secreted by bacteria can degrade the proteins into nitrogen-containing small molecules that cause food spoilage. Seven serine protease and six metalloprotease genes were identified in the GWT 902 and GWT 904 genomes, respectively (Table 4). Serine proteases are the major extracellular proteases of *Pseudomonas psychrophila* and *S. putrefaciens*, which can degrade myofibrillar proteins, causing severe protein degradation in aquatic products [41]. A metalloproteinase of the M23 family identified in *S. putrefaciens* has been reported to degrade fish myofibrillar proteins and sarcoplasmic proteins [46]. In addition, genes encoding ATP-dependent Clp proteases were identified in two bacteria [47,48]. The identification of genes encoding lipases and proteases indicates that two strains have the potential to degrade lipids and proteins in seafood.

### 3.11. Quorum Sensing System

QS is an intercellular communication process by which bacteria regulate population behavior by secreting signaling molecules. In GWT 902 and GWT 904, genes associated with LuxS/AI-2-type QS system (*luxS*, *lsrB*, *lsrC*, *lsrD*, *lsrA*, *lsrK*, *lsrR*, *lsrG*) were annotated (Table 5). The *luxS* gene encodes S-ribosylhomocysteine lyase, which is responsible for the generation of 4,5-dihydroxy-2,3-pentanedione (DPD), the AI-2 precursor [49,50]. Extracellular AI-2 binds to the receptor protein encoded by the *lsrB* gene and is internalized by the cell through transporter proteins encoded by *lsrCD*. Intracellularly, AI-2 is phosphorylated by the kinase LsrK, thereby inactivating the transcriptional repressor LsrR and activating downstream gene expression [51]. In addition, *qseB* gene encoding response regulator protein QseB and *qseC* gene encoding histidine kinase QseC, which sense AI-3-type QS signaling molecules, were identified in GWT 902 and GWT 904. QseB/QseC is a two-component system which is involved in the regulation of multiple bacterial behaviors, such as flagella and motility, antibiotic resistance, and biofilm formation [52].

The LuxS/AI-2 QS system has been reported to regulate various bacterial physiological functions, including biofilm formation, virulence factor expression, and antibiotic resistance [53,54,55]. Moreover, LuxS not only serves as the key enzyme for AI-2 biosynthesis but also plays an essential role in activated methyl cycle (AMC), affecting multiple metabolic pathways [49,56]. Learman et al. [57] demonstrated that LuxS influences biofilm formation through AMC, and is also essential for the metabolism of methionine in *Shewanella oneidensis*. Hu et al. [50] further confirmed that *luxS* deletion in *S. putrefaciens* significantly reduced H_2_S production, diminished biofilm formation capacity, and decreased TVB-N accumulation in fish homogenates, while the exogenous addition of DPD and key circulating substances of AMC effectively alleviated the effects of *luxS* deletion. Therefore, we hypothesized that this QS system may be involved in regulating bacterial behaviors such as biofilm formation, spoilage activity, sulfur metabolism, and antibiotic resistance in GWT 902 and GWT 904.

### 3.12. Adaptation to Stress

The ability of microorganisms to adapt and survive in various stressful environments is critical for their growth and behavior. A series of stress resistance genes of GWT 902 and GWT 904 are shown in Table 5. A total of 7 and 9 genes encoding cold shock proteins (Csps) were identified in GWT 902 and GWT 904, respectively. Csps play a pivotal role in bacterial temperature adaptation, with CspA serving as the major cold shock protein in *Escherichia coli* [58]. Additionally, certain Csps can also be induced under a variety of stress conditions [59,60,61]. Both strains possess ion transport systems that are crucial for osmotic stress response. GWT 902 and GWT 904 contained four and five genes encoding sodium/proton antiporter, respectively. Other related genes that maintain the dynamic balance of ions, such as *trkA* and *trkH* encoding Trk system potassium transporter, *corA* and *corC* encoding magnesium/cobalt transporter, *kefB* and *kefG* encoding glutathione-regulated potassium-efflux system protein, were also identified. Moreover, genes associated with the general stress response were identified in both strains, including those encoding alkyl hydroperoxide reductase and stringent starvation protein [62,63]. Remarkably, *rpoS* and *rpoN* are identified in GWT 902 and GWT 904, which have been reported as typical environmental response regulators involved in bacterial stress survival [64]. *rpoS* has been shown to regulate protease secretion and degradation activities in *S. baltica* and *Pseudomonas fluorescens* [65,66]. *rpoN* plays an important role in bacterial swimming motility, biofilm formation, stress, and antibiotic resistance by modulating the expression of a large number of genes [67]. The presence of these genes is likely to facilitate adaptation to environmental stress in *M. psychrotolerans* and *M. sibonii*.

### 3.13. Drug Resistance

Bacterial drug resistance poses a major challenge to food safety and human health. The CARD database contains information of antibiotic resistance genes, related proteins, and antibiotic resistance mechanisms [68]. The genomes of the two strains were annotated with 261 and 266 antibiotic-related genes in the CARD database, which were mainly resistance genes of tetracyclines, fluoroquinolone, peptide, macrolide, and penam (Table 6). Tetracycline resistance has been reported to be widespread in *M. sibonii*, and it has also been reported in *M. psychrotolerans* isolated from rainbow trout (*Oncorhynchus mykiss*) [69,70]. Notably, 26 and 30 carbapenem resistance-associated genes were identified in GWT 902 and GWT 904, respectively (Table 6). Carbapenem resistance in these two strains may develop through multiple mechanisms, including carbapenemase synthesis, alterations in penicillin-binding proteins, and efflux pump systems [71]. Bacterial resistance to carbapenem antibiotics has emerged as one of the most critical public health concerns worldwide [72]. The bacterial multidrug efflux system is the main resistance mechanism of bacteria [73]. AcrAB–TolC efflux pump-related genes, such as *ramA* and *marA*, were identified in two strains. The AcrAB–TolC efflux pump expels antibiotics and other antimicrobial drugs with the help of membrane fusion proteins, enabling bacteria to develop antibiotic resistance [74]. AcrAB efflux pumps were reported to be associated with resistance to tigecycline in *M. morganii* and *M. sibonii* [39,75]. In addition, nitrofuran and streptogramin B antibiotic genes were only found in GWT 902, whereas polyamine antibiotic genes were only found in GWT 904.

## 4. Conclusions

In this study, two histamine-producing psychrotrophic *Morganella* strains, GWT 902 and GWT 904, were isolated from yellowfin tuna. Phenotypic characterization reveals differences in growth temperature, NaCl tolerance, and D-galactose fermentation. Further analysis of histamine production capacity demonstrates that GWT 902 exhibited higher histamine accumulation at 4 °C. ANI analysis classified GWT 902 as *M. psychrotolerans* and GWT 904 as *M. sibonii*. Gene function analysis identified the presence of histidine decarboxylase gene clusters in both strains. The HDC (378 amino acids) shared 95.24% sequence identity, with lysine residues at the active site conserved. However, amino acid variations in other sites may account for the differences in histamine production between the two strains. Genes associated with putrescine production, sulfur metabolism, and protease and lipase secretion were identified in both strains, indicating their spoilage potential in seafood. The identification of QS system-related genes suggested a regulatory role in bacterial behavior, though the specific role of QS in these strains requires further exploration. Additionally, genes related to stress adaptation and antibiotic resistance were identified, suggesting their ability to survive under various environmental stresses and potential risks to food safety. This study examined the phenotypic and genomic differences between *M. psychrotolerans* and *M. sibonii*, thereby enhancing our understanding of psychrotrophic histamine producers. The genomic information, including histidine decarboxylase gene clusters, QS-related genes, and other spoilage-related genes, may provide potential targets for inhibiting bacterial growth and addressing the quality and safety issues associated with seafood.

## Figures and Tables

**Table 1:** Characteristics of GWT 902 and GWT 904.

Characteristics	Strains	
	GWT 902	GWT 904
2 °C	+	-
4 °C	+	+
8.5% NaCl	-	+
D-galactose	-	+

Key: “+” stands for growth or positive; “-” stands for no growth or negative.

**Table 2:** Histamine production of GWT 902 and GWT 904.

Strains	Histamine Content (mg/L)	
	20 °C, 60 h	4 °C, 8 Days
GWT 902	4807.79 ± 315.40 ^a^	4708.17 ± 113.30 ^a^
GWT 904	5590.27 ± 595.08 ^a^	3731.57 ± 399.72 ^b^

Different letters indicate significant differences between the two strains at the same temperature (*p* < 0.05).

**Table 3:** Genome features of GWT 902 and GWT 904.

Genome Features	Strains	
	GWT 902	GWT 904
Genome size (bp)	3,990,716	4,200,263
DNA G + C (%)	47.97	50.39
Number of CDSs	3608	3946
Number of rRNA genes	22	22
Number of tRNA genes	77	81

**Table 4:** Spoilage-related pathways and genes of GWT 902 and GWT 904.

Spoilage-Related Pathways	Encoded Protein	Gene	Gene ID	
			GWT 902	GWT 904
Histamine metabolism	Putative histidine–histamine antiporter	*hdcT1*	gene3203	gene3565
	Histidine decarboxylase	*hdc*	gene3204	gene3566
	Putative histidine–histamine antiporter	*hdcT2*	gene3205	gene3567
	Histidyl-tRNA synthetase	*his* *RS*	gene3206	gene3568
Putrescine metabolism	Arginine decarboxylase	*speA*	gene2708	gene2989
	Agmatinase	*speB*	gene2707	gene2988
	Ornithine decarboxylase	*speC*	gene1271	gene1319
	Putrescine importer	*puuP*	gene1075 gene2433	gene1138 gene2591
	Spermidine/putrescine transport system ATP-binding protein Spermidine/putrescine ABC transporter permease	*potA*	gene3252	gene3623
		*potB*	gene3251	gene3622
	Spermidine/putrescine ABC transporter permease	*potC*	gene3250	gene3621
	Spermidine/putrescine ABC transporter substrate-binding protein	*potD*	gene3249	gene2899
Sulfur metabolism	Cysteine synthase	*cysM*	gene2325	gene1482
	Cysteine synthase	*cysK*	gene2535	gene2732
	Sulfate/thiosulfate transport system substrate-binding protein	*cysP*	gene2329	gene1478
	Sulfate/thiosulfate transport system permease protein	*cysU*	gene2328	gene1479
	Sulfate/thiosulfate transport system permease protein	*cysW*	gene2327	gene1480
	Sulfate/thiosulfate transport system ATP-binding protein	*cysA*	gene2326	gene1481
	Sulfate transport protein	*cysZ*	gene2534	gene2731
	Assimilatory sulfite reductase (NADPH) hemoprotein subunit	*cysI*	-	gene3339
	NADPH-dependent assimilatory sulfite reductase flavoprotein subunit	*cysJ*	-	gene3340
	3′(2′), 5′-bisphosphate nucleotidase	*cysQ*	gene3425	gene3778
	Serine O-acetyltransferase	*cysE*	gene3641	gene3982
	Sulfide: quinone oxidoreductase	*sqr*	gene0547	gene0619
	Taurine dioxygenase	*tauD*	gene3000	gene3344
	Tetrathionate reductase subunit TtrA	*ttrA*	gene2301	gene1518
	Tetrathionate reductase subunit TtrB	*ttrB*	gene2303	gene1516
	Tetrathionate reductase subunit TtrC	*ttrC*	gene2302	gene1517
	Thiosulfate sulfurtransferase	*glpE*	gene0143	gene0136
	Thiosulfate/3-mercaptopyruvate sulfurtransferase	*sseA*	gene1063	gene1130
Lipase	Lipoyl synthase	*lipA*	gene0940	gene1011
	Lipoyl(octanoyl) transferase	*lipB*	gene0941	gene1012
	Lysophospholipase	*pldB*	gene3457	gene3807
	Esterase FrsA	*frsA*	gene0879	gene0927
	Esterase	*ybfF*	-	gene1057
Protease	Serine protease DegQ	*degQ*	gene2999	gene3338
	Serine protease DegS	*degS*	gene3001	gene3345
	Rhomboid protease GluP	*gluP*	gene1100	gene1168
	Rhomboid protease GlpG	*glpG*	gene0142 gene1977	gene0135 gene2029
	Serine protease inhibitor ecotin	*eco*	gene2573	gene2850
	SprT family zinc-dependent metalloprotease	*sprT*	gene2714	gene2994
	Metalloprotease PmbA	*pmbA*	gene3022	gene3366
	Metalloprotease	*rseP*	gene0521	gene0651
	CPBP family intramembrane metalloprotease	*-*	gene1026	gene1097
	Metalloprotease TldD	*tldD*	gene3026	gene3370
	Cell division protease FtsH	*ftsH*	gene3403	gene3754
	ATP-dependent Clp protease ATP-binding subunit ClpB	*clpB*	gene0913	gene0960
	ATP-dependent Clp protease adaptor protein ClpS	*clpS*	gene1137	gene1210
	ATP-dependent Clp protease ATP-binding subunit ClpA	*clpA*	gene1138	gene1211
	ATP-dependent Clp protease ATP-binding subunit ClpX	*clpX*	gene2889	gene3161
	ATP-dependent Clp protease, protease subunit	*clpP*	gene2890	gene0349 gene3162

**Table 5:** Genes associated with QS system and adaption to stress of GWT 902 and GWT 904.

	Encoded Protein	Gene	Gene ID	
			GWT 902	GWT 904
QS system	S-ribosylhomocysteine lyase	*luxS*	gene0896	gene0944
	Autoinducer 2 ABC transporter substrate-binding protein	*lsrB*	gene1867	gene1923
	(4S)-4-hydroxy-5-phosphonooxypentane-2,3-dione isomerase	*lsrG*	gene1865	gene1921
	Autoinducer-2 kinase	*lsrK*	gene1872	gene1928
	Autoinducer 2 ABC transporter ATP-binding protein	*lsrA*	gene1870	gene1926
	Autoinducer 2 ABC transporter permease	*lsrC*	gene1869	gene1925
	Autoinducer 2 ABC transporter permease	*lsrD*	gene1868	gene1924
	3-hydroxy-5-phosphonooxypentane-2,4-dione thiolase	*lsrF*	gene1866	gene1922
	Transcriptional regulator	*lsrR*	gene1871	gene1927
Adaptation to stress	Cold shock protein	*cspA*	gene0938 gene1136 gene1162 gene1463 gene1584 gene2292 gene2461	gene1009 gene1209 gene1242 gene1529 gene1715 gene2356 gene2413 gene2660 gene2667
	Sodium/proton antiporter NhaB	*nhaB*	gene1725	gene2229
	Na^+^/H^+^ antiporter	*nhaK*	gene0324 gene0815	gene0389 gene1176
	Na^+^/H^+^ antiporter	*nhaA*	gene0427	gene0497 gene1033
	Trk system potassium transporter	*trkA*	gene3527	gene3874
	Trk system potassium transporter	*trkH*	gene0346	gene0414
	Magnesium/cobalt transporter CorA	*corA*	gene3461	gene3811
	Magnesium/cobalt transporter CorC	*corC*	gene0968	gene1037
	Glutathione-regulated potassium-efflux system protein KefB	*kefB*	gene0238	gene0245
	Glutathione-regulated potassium-efflux system ancillary protein KefG	*kefG*	gene0237	grnr0244
	RNA polymerase sigma factor	*rpoS*	gene0561	gene0633
	Sigma-54 RNA polymerase factor sigma-54	*rpoN*	gene3015	gene3359
	NADH-dependent peroxiredoxin subunit C	*ahpC*	gene3253	gene3625
	NADH-dependent peroxiredoxin subunit F	*ahpF*	gene3254	gene3626
	Stringent starvation protein A	*sspA*	gene2994	gene3333
	Stringent starvation protein B	*sspB*	gene2993	gene3332

**Table 6:** Classification of drug resistance genes in GWT 902 and GWT 904.

Drug Class	Number of Genes	
	GWT 902	GWT 904
Tetracycline antibiotic	58	61
Fluoroquinolone antibiotic	52	50
Penam	34	43
Cephalosporin	33	41
Peptide antibiotic	36	38
Macrolide antibiotic	36	37
Disinfecting agents and antiseptics	28	33
Cephamycin	25	31
Phenicol antibiotic	33	31
Carbapenem	26	30

## Data Availability

The original contributions presented in this study are included in the article. Further inquiries can be directed to the corresponding authors.

## References

[1] Omer A.K., Mohammed R.R., Ameen P.S.M., Abas Z.A., Ekici K. (2021). Presence of Biogenic Amines in Food and Their Public Health Implications: A Review. J. Food Prot..

[2] Durak-Dados A., Michalski M., Osek J. (2020). Histamine and Other Biogenic Amines in Food. J. Vet. Res..

[3] DeBEER J., Bell J.W., Nolte F., Arcieri J., Correa G. (2021). Histamine Limits by Country: A Survey and Review. J. Food Prot..

[4] Landete J.M., De las Rivas B., Marcobal A., Muñoz R. (2008). Updated Molecular Knowledge about Histamine Biosynthesis by Bacteria. Crit. Rev. Food Sci. Nutr..

[5] Visciano P., Schirone M., Paparella A. (2020). An Overview of Histamine and Other Biogenic Amines in Fish and Fish Products. Foods.

[6] Nevado D.L., Delos Santos S., Bastian G., Deyta J., Managuelod E., Fortaleza J.A., De Jesus R. (2023). Detection, Identification, and Inactivation of Histamine-Forming Bacteria in Seafood: A Mini-Review. J. Food Prot..

[7] Bonnin R.A., Creton E., Perrin A., Girlich D., Emeraud C., Jousset A.B., Duque M., Jacquemin A., Hopkins K., Bogaerts P. (2024). Spread of Carbapenemase-Producing Morganella Spp from 2013 to 2021: A Comparative Genomic Study. Lancet Microbe.

[8] Emborg J., Dalgaard P. (2008). Growth, Inactivation and Histamine Formation of *Morganella psychrotolerans* and *Morganella morganii* —Development and Evaluation of Predictive Models. Int. J. Food Microbiol..

[9] Wang D., Yamaki S., Kawai Y., Yamazaki K. (2020). Histamine Production Behaviors of a Psychrotolerant Histamine-Producer, *Morganella psychrotolerans*, in Various Environmental Conditions. Curr. Microbiol..

[10] Emborg J., Ahrens P., Dalgaard P. (2007). *Morganella psychrotolerans*—Identification, Histamine Formation and Importance for Histamine Fish Poisoning. Ph.D. Thesis.

[11] Zhang R., Yang T., Zhang Q., Liu D., Elhadidy M., Ding T. (2022). Whole-Genome Sequencing: A Perspective on Sensing Bacterial Risk for Food Safety. Curr. Opin. Food Sci..

[12] Wells J.M., Bennik M.H.J. (2003). Genomics of Food-Borne Bacterial Pathogens. Nutr. Res. Rev..

[13] Shelburne S.A., Kim J., Munita J.M., Sahasrabhojane P., Shields R.K., Press E.G., Li X., Arias C.A., Cantarel B., Jiang Y. (2017). Whole-Genome Sequencing Accurately Identifies Resistance to Extended-Spectrum β-Lactams for Major Gram-Negative Bacterial Pathogens. Clin. Infect. Dis..

[14] Palmieri N., Hess C., Hess M., Alispahic M. (2020). Sequencing of Five Poultry Strains Elucidates Phylogenetic Relationships and Divergence in Virulence Genes in *Morganella morganii*. BMC Genom..

[15] Li J., Wang D., Chen S., Wu Y., Li C., Wang Y. (2024). Contamination of *Morganella psychrotolerans* in Fish Products and Histamine Production Capacity of the Isolated Strains. Food Sci..

[16] Drancourt M., Bollet C., Carlioz A., Martelin R., Gayral J.-P., Raoult D. (2000). 16S Ribosomal DNA Sequence Analysis of a Large Collection of Environmental and Clinical Unidentifiable Bacterial Isolates. J. Clin. Microbiol..

[17] Emborg J., Dalgaard P., Ahrens P. (2006). *Morganella psychrotolerans* Sp. Nov., a Histamine-Producing Bacterium Isolated from Various Seafoods. Int. J. Syst. Evol. Microbiol..

[18] Wick R.R., Judd L.M., Gorrie C.L., Holt K.E. (2017). Unicycler: Resolving Bacterial Genome Assemblies from Short and Long Sequencing Reads. PLoS Comput. Biol..

[19] Walker B.J., Abeel T., Shea T., Priest M., Abouelliel A., Sakthikumar S., Cuomo C.A., Zeng Q., Wortman J., Young S.K. (2014). Pilon: An Integrated Tool for Comprehensive Microbial Variant Detection and Genome Assembly Improvement. PLoS ONE.

[20] Besemer J., Lomsadze A., Borodovsky M. (2001). GeneMarkS: A Self-Training Method for Prediction of Gene Starts in Microbial Genomes. Implications for Finding Sequence Motifs in Regulatory Regions. Nucleic Acids Res..

[21] Lowe T.M., Eddy S.R. (1997). tRNAscan-SE: A Program for Improved Detection of Transfer RNA Genes in Genomic Sequence. Nucleic Acids Res..

[22] Stothard P., Wishart D.S. (2005). Circular Genome Visualization and Exploration Using CGView. Bioinformatics.

[23] Zhou W., Gao S., Zheng J., Zhang Y., Zhou H., Zhang Z., Cao X., Shen H. (2022). Identification of an *Aerococcus urinaeequi* Isolate by Whole Genome Sequencing and Average Nucleotide Identity Analysis. J. Glob. Antimicrob. Resist..

[24] Emms D.M., Kelly S. (2019). OrthoFinder: Phylogenetic Orthology Inference for Comparative Genomics. Genome Biol..

[25] Bjornsdottir-Butler K., Bowers J.C., Benner R.A. (2015). Prevalence and Characterization of High Histamine-Producing Bacteria in Gulf of Mexico Fish Species. J. Food Prot..

[26] Goris J., Konstantinidis K.T., Klappenbach J.A., Coenye T., Vandamme P., Tiedje J.M. (2007). DNA–DNA Hybridization Values and Their Relationship to Whole-Genome Sequence Similarities. Int. J. Syst. Evol. Microbiol..

[27] Kanehisa M., Goto S. (2000). KEGG: Kyoto Encyclopedia of Genes and Genomes. Nucleic Acids Res..

[28] Kanehisa M., Goto S., Sato Y., Kawashima M., Furumichi M., Tanabe M. (2014). Data, Information, Knowledge and Principle: Back to Metabolism in KEGG. Nucl. Acids Res..

[29] Koonin E.V. (2005). Orthologs, Paralogs, and Evolutionary Genomics. Annu. Rev. Genet..

[30] Medini D., Donati C., Tettelin H., Masignani V., Rappuoli R. (2005). The Microbial Pan-Genome. Curr. Opin. Genet. Dev..

[31] Li H., Zhu J., Xiao Y., Zhang S., Sun Y., Liu Z., Chu C., Hu X., Yi J. (2023). Biodiversity of Lactic Acid Bacteria in Traditional Fermented Foods in Yunnan Province, China, and Comparative Genomics of Lactobacillus plantarum. Fermentation.

[32] Carr V.R., Shkoporov A., Hill C., Mullany P., Moyes D.L. (2021). Probing the Mobilome: Discoveries in the Dynamic Microbiome. Trends Microbiol..

[33] Vo J.L., Martínez Ortiz G.C., Subedi P., Keerthikumar S., Mathivanan S., Paxman J.J., Heras B. (2017). Autotransporter Adhesins in Escherichia Coli Pathogenesis. Proteomics.

[34] Lingzhi L., Haojie G., Dan G., Hongmei M., Yang L., Mengdie J., Chengkun Z., Xiaohui Z. (2018). The Role of Two-Component Regulatory System in β-Lactam Antibiotics Resistance. Microbiol. Res..

[35] Papon N., Stock A.M. (2019). Two-Component Systems. Curr. Biol..

[36] Sultan M., Arya R., Kim K.K. (2021). Roles of Two-Component Systems in Pseudomonas aeruginosa Virulence. Int. J. Mol. Sci..

[37] Vaaler G.L., Snell E.E. (1989). Pyridoxal 5’-Phosphate Dependent Histidine Decarboxylase: Overproduction, Purification, Biosynthesis of Soluble Site-Directed Mutant Proteins, and Replacement of Conserved Residues. Biochemistry.

[38] Ferrario C., Borgo F., de las Rivas B., Muñoz R., Ricci G., Fortina M.G. (2014). Sequencing, Characterization, and Gene Expression Analysis of the Histidine Decarboxylase Gene Cluster of *Morganella morganii*. Curr. Microbiol..

[39] Ryser L.T., Arias-Roth E., Perreten V., Irmler S., Bruggmann R. (2021). Genetic and Phenotypic Diversity of *Morganella morganii* Isolated From Cheese. Front. Microbiol..

[40] Remenant B., Jaffrès E., Dousset X., Pilet M.-F., Zagorec M. (2015). Bacterial Spoilers of Food: Behavior, Fitness and Functional Properties. Food Microbiol..

[41] Jia S., Jia Z., An J., Ding Y., Chang J., Wang Y., Zhou X. (2024). Insights into the Fish Protein Degradation Induced by the Fish-Borne Spoiler *Pseudomonas psychrophila* and *Shewanella putrefaciens*: From Whole Genome Sequencing to Quality Changes. Int. J. Food Microbiol..

[42] Wang X.-Y., Yan J., Xie J. (2023). Applications of Genomics, Metabolomics, Fourier Transform Infrared in the Evaluation of Spoilage Targets of *Shewanella putrefaciens* from Spoiled Bigeye Tuna. J. Agric. Food Chem..

[43] Li J., Yu H., Yang X., Dong R., Liu Z., Zeng M. (2020). Complete Genome Sequence Provides Insights into the Quorum Sensing-Related Spoilage Potential of *Shewanella baltica* 128 Isolated from Spoiled Shrimp. Genomics.

[44] Motohashi H., Akaike T. (2019). Sulfur-Utilizing Cytoprotection and Energy Metabolism. Curr. Opin. Physiol..

[45] Abril A.G., Calo-Mata P., Villa T.G., Böhme K., Barros-Velázquez J., Sánchez-Pérez Á., Pazos M., Carrera M. (2024). Comprehensive Shotgun Proteomic Characterization and Virulence Factors of Seafood Spoilage Bacteria. Food Chem..

[46] Yi Z., Yan J., Ding Z., Xie J. (2023). Purification and Characterizations of a Novel Extracellular Protease from *Shewanella putrefaciens* Isolated from Bigeye Tuna. Food Biosci..

[47] Chandu D., Nandi D. (2004). Comparative Genomics and Functional Roles of the ATP-Dependent Proteases Lon and Clp during Cytosolic Protein Degradation. Res. Microbiol..

[48] Dong S., Chen H., Zhou Q., Liao N. (2021). Protein Degradation Control and Regulation of Bacterial Survival and Pathogenicity: The Role of Protein Degradation Systems in Bacteria. Mol. Biol. Rep..

[49] Winzer K., Hardie K.R., Williams P. (2003). LuxS and Autoinducer-2: Their Contribution to Quorum Sensing and Metabolism in Bacteria. Adv. Appl. Microbiol..

[50] Hu Z., Chin Y., Yuan C., Ge Y., Hang Y., Wang D., Yao Q., Hu Y. (2024). The luxS Deletion Reduces the Spoilage Ability of *Shewanella putrefaciens*: An Analysis Focusing on Quorum Sensing and Activated Methyl Cycle. Food Microbiol..

[51] Meng F., Zhao M., Lu Z. (2022). The LuxS/AI-2 System Regulates the Probiotic Activities of Lactic Acid Bacteria. Trends Food Sci. Technol..

[52] Zhu Y., Dou Q., Du L., Wang Y. (2023). QseB/QseC: A Two-Component System Globally Regulating Bacterial Behaviors. Trends Microbiol..

[53] Wang Y., Wang Y., Sun L., Grenier D., Yi L. (2018). The LuxS/AI-2 System of *Streptococcus suis*. Appl. Microbiol. Biotechnol..

[54] Yang Q., Wang Y., An Q., Sa R., Zhang D., Xu R. (2021). Research on the Role of LuxS/AI-2 Quorum Sensing in Biofilm of *Leuconostoc citreum* 37 Based on Complete Genome Sequencing. 3 Biotech.

[55] Wang Y., Liu B., Grenier D., Yi L. (2019). Regulatory Mechanisms of the LuxS/AI-2 System and Bacterial Resistance. Antimicrob. Agents Chemother..

[56] Doherty N., Holden M.T.G., Qazi S.N., Williams P., Winzer K. (2006). Functional Analysis of *luxS* in *Staphylococcus aureus* Reveals a Role in Metabolism but Not Quorum Sensing. J. Bacteriol..

[57] Learman D.R., Yi H., Brown S.D., Martin S.L., Geesey G.G., Stevens A.M., Hochella M.F. (2009). Involvement of *Shewanella oneidensis* MR-1 LuxS in Biofilm Development and Sulfur Metabolism. Appl. Environ. Microbiol..

[58] Jiang W., Hou Y., Inouye M. (1997). CspA, the Major Cold-Shock Protein of Escherichia coli, Is an RNA Chaperone. J. Biol. Chem..

[59] Phadtare S. (2004). Recent Developments in Bacterial Cold-Shock Response. Curr. Issues Mol. Biol..

[60] Ray S., Da Costa R., Thakur S., Nandi D. (2020). Salmonella Typhimurium Encoded Cold Shock Protein E Is Essential for Motility and Biofilm Formation. Microbiology.

[61] Muchaamba F., von Ah U., Stephan R., Stevens M.J.A., Tasara T. (2022). Deciphering the Global Roles of Cold Shock Proteins in *Listeria monocytogenes* Nutrient Metabolism and Stress Tolerance. Front. Microbiol..

[62] Rocha E.R., Smith C.J. (1999). Role of the Alkyl Hydroperoxide Reductase (ahpCF) Gene in Oxidative Stress Defense of the Obligate Anaerobe Bacteroides Fragilis. J. Bacteriol..

[63] Hansen A.-M., Gu Y., Li M., Andrykovitch M., Waugh D.S., Jin D.J., Ji X. (2005). Structural Basis for the Function of Stringent Starvation Protein A as a Transcription Factor. J. Biol. Chem..

[64] Wang D., Cui F., Ren L., Tan X., Lv X., Li Q., Li J., Li T. (2022). Complete Genome Analysis Reveals the Quorum Sensing-Related Spoilage Potential of *Pseudomonas fluorescens* PF08, a Specific Spoilage Organism of Turbot (Scophthalmus Maximus). Front. Microbiol..

[65] Feng L., Bi W., Chen S., Zhu J., Liu X. (2021). Regulatory Function of Sigma Factors RpoS/RpoN in Adaptation and Spoilage Potential of *Shewanella baltica*. Food Microbiol..

[66] Liu X., Ji L., Wang X., Li J., Zhu J., Sun A. (2018). Role of RpoS in Stress Resistance, Quorum Sensing and Spoilage Potential of *Pseudomonas fluorescens*. Int. J. Food Microbiol..

[67] Liu X., Ye Y., Zhu Y., Wang L., Yuan L., Zhu J., Sun A. (2021). Involvement of RpoN in Regulating Motility, Biofilm, Resistance, and Spoilage Potential of *Pseudomonas fluorescens*. Front. Microbiol..

[68] Alcock B.P., Raphenya A.R., Lau T.T.Y., Tsang K.K., Bouchard M., Edalatmand A., Huynh W., Nguyen A.-L.V., Cheng A.A., Liu S. (2020). CARD 2020: Antibiotic Resistome Surveillance with the Comprehensive Antibiotic Resistance Database. Nucleic Acids Res..

[69] Stock I., Wiedemann B. (1998). Identification and Natural Antibiotic Susceptibility of *Morganella morganii*. Diagn. Microbiol. Infect. Dis..

[70] Oh W.T., Jun J.W., Giri S.S., Yun S., Kim H.J., Kim S.G., Kim S.W., Kang J.W., Han S.J., Kwon J. (2020). *Morganella psychrotolerans* as a Possible Opportunistic Pathogen in Rainbow Trout (Oncorhynchus Mykiss) Fisheries. Aquaculture.

[71] Ma J., Song X., Li M., Yu Z., Cheng W., Yu Z., Zhang W., Zhang Y., Shen A., Sun H. (2023). Global Spread of Carbapenem-Resistant *Enterobacteriaceae*: Epidemiological Features, Resistance Mechanisms, Detection and Therapy. Microbiol. Res..

[72] Aurilio C., Sansone P., Barbarisi M., Pota V., Giaccari L.G., Coppolino F., Barbarisi A., Passavanti M.B., Pace M.C. (2022). Mechanisms of Action of Carbapenem Resistance. Antibiotics.

[73] Zhang F., Cheng W. (2022). The Mechanism of Bacterial Resistance and Potential Bacteriostatic Strategies. Antibiotics.

[74] Weston N., Sharma P., Ricci V., Piddock L.J.V. (2018). Regulation of the AcrAB-TolC Efflux Pump in Enterobacteriaceae. Res. Microbiol..

[75] Ruzin A., Keeney D., Bradford P.A. (2005). AcrAB Efflux Pump Plays a Role in Decreased Susceptibility to Tigecycline in *Morganella morganii*. Antimicrob. Agents Chemother..

